# Address at the Opening of the Session of 1896-97

**Published:** 1896-12

**Authors:** James B. Hodgkin

**Affiliations:** Professor of Dental Pathology, etc., in the Medical and Dental Department of Howard University


### THE DENTAL REGISTER.

VOL. L.] DECEMBER, 1896. [No. 12.

### PROCEEDINGS.

### Address at the Opening of the Session of 1896-7.

BY JAMES B. HODGKIN, D.D.S.

Professor of Dental Pathology, etc., in the Medical and Dental Department of Howard University.

*Ladies and Gentlemen:*

It has been my fortune to make addresses, not a few, in the course of my professional career, but it has chanced that this is the first time I have stood in precisely the position I now occupy. Not that I have not done a deal of talking, more possibly than was good for those who heard me, but, then, most of these hearers were students and did not know any better, or found it best not to try to escape from the assaults I made on their ears. Be this as it may, I think it best, just now, to be a little careful, not only of what I say, but of the time I occupy, for time, I believe, is about the only possession, which, once lost, we can never retrieve.

Fortunately for me, I have about me some certain grave and reverend seigniors whose sympathy I am sure I have; they, or at all events, most of them, having tried this "address business" before. They know, possibly to their sorrow, how difficult it is to tell an old story in a new way.

And I am sure I shall earn the gratitude of, at least, some who are present if I am brief—some who will hear from me, in a more or less tedious way, in the next six months or so. For you will hear about bones, arteries, tendons, muscles—the histologic anatomy of the soul almost—much that to the freshman is new, startling, seemingly impossible. Yes, you will not only hear this, but you will get to delight in it, revel in the butcheries of the dead-house, and have, I fear, little compassion even for the poor fellow that is dying if you can be "in at the autopsy."

For such is the student's life. Himself, possibly, the most sympathetic of men as he stands at the bedside and watches the death-struggle in the fevered patient who is feebly battling for a few more hours of life, or, it may be, tying the severed artery that is so rapidly leaking away the life of the wounded man; he seems to be obliged to get in the hospital and the dissecting-room that he may be in sympathy with the sufferer in the hospital cot, or maimed and mangled in the horrible railway collision. Of such seeming impossibilities is the warp and woof of our life made. It seems strange, indeed, but it seems to me that the hard and the soft, the resolute and the tender are so inextricably mixed and mingled in our life as to be incapable of separation.

Who, but a hard and cruel man, can relentlessly thrust a knife into an inflamed and diseased organ? Who, but the most unsympathetic man, can be ready to do such work?

I think I have wandered a little from what I started out to say. It was, that the training we get here should make us not only much wiser, but much better men and women. That the schooling we get here in witnessing of human suffering, if it works out its proper results, should make us more tolerant of other people's complaints, more forbearing of their groans and cries. If the life-study we get here, in witnessing suffering does not help us to be more patient with querulous sickness, and invalidism only active in groans and complaints, we will, as I take it, fail to have learned one of the chief lessons of the student. To be calm in the presence of the discontented and alarmed, to allay the fears arising from ignorance, to be so helpful in mien and port before those who are all fears and gloom—this is no small part of the learning we are to acquire.

Now, I will tell a little secret. It is that of those whose fortune it has been to have fallen into my hands for the minor surgery of the teeth, the most timid patients I have had have been doctors. Somehow, the constant witnessing of suffering does not bring ability to bear it, nor does the skill to cure the sick and maimed give nerve to take suffering for one's self with fortitude. I have no time to analyze this interesting puzzle, but it is a fact, as most of my dental friends will testify.

I once said that, at least so far as a dental infirmary was concerned, that the motto over the gates of the Inferno might, with a slight interpolation, be placed over the door: "He who enters here leaves hope (of teeth) behind." For your average dental student, and I take it that the average medical student is not unlike him, has not the keen appreciation he might have of the fact that the poor, penniless wretch before him, with aching tooth and swollen jaw, is flesh and blood, with nerves as sensitive and tissues as shrinking from pain as the well-to-do office patient. And I beg you to recollect and keep constantly in mind the fact that the hospital patient is a real live man, or woman, or child, with all the dread of physical suffering that you have.

I realize how difficult is the situation here. To be gently severe; to be pitilessly tender; these seem opposites and antagonistics. Yet, I do think they can be blended, and the firm hand holding the keen knife can be firm, yet tender. "The eye is the window of the soul," and your patient, looking in your face will read there, as in an open book, whether you feel for him and will spare him so far as good surgery will admit.

I hear one whisper to another, "This is an old fogy, and this is something written before the days of ether or chloroform.' Not so; what has been will be, and no anæsthetic yet discovered can take away the terror, or the dreadful fear. Lie down to-night, close your eyes and calm yourself with the thought that you may never wake again, or that, possibly, during your sleep you will be cut and maimed and mangled and mutilated in the interests of science, and not even the idea of future health as contrasted with present suffering will make you comfortable or drowsy. Blessed be the anæsthetic, but there is terror withal, an intuitive horror felt even by the most savage people at the mutilation of this casket of the soul, this divinely immortal body.

But I have, I dare say, said enough on this subject, and only repeat that I hope you will daily study, among these poor unfortunates who are doubly so in being diseased and in being obliged to come here, the fine art of sympathy. It need not unsteady the hand, or make less firm the will, but it will be a precious balsam to those who are your victims by being the victim of disease.

Being here to receive the truth, I trust a word on the manner of its reception may not be amiss. In that old book, which contains a great deal of truth despite the assaults made on it by modern unbelievers, there is a saying something like this: That unless you receive the truth as little children do it will take no hold on your minds or hearts. Now, little children believe what is told them because they do not know any better; they receive the truth they hear into virgin soil, nothing doubting that it is *the* truth. What I wish to say is that if you, coming here to learn, will train your minds (and I may say your hearts as well), simply to believe what you hear in these lecture-rooms, nothing doubting, it will be a most wonderful way to facilitate your education. To hear, and then to doubt; to listen, and then to be dubious as to the doctrine, may be the role of the philosopher, but not that of the student. You have no time for such. To be constantly in doubt as to whether the lecturer is correct in his statement, and to make a mental reservation, going to the textbooks to verify or disprove what has been taught is plainly a waste of time as well as of effort. And yet, I have known such students.

If *education* is to draw out what is in us (and the ancients evidently thought so when they adopted a word taken from the mechanical arts), e-duco, wire-pulling. (I beg that you will not associate this, in any way, with politics.) It involves the idea of ductility, or, so to speak, plasticity, on the part of the thing drawn, and a doubting mind is like a stiffening alloy in the pure metal, making the e-duco difficult, perchance, impossible. So I beg you at the very start to throw away all pronounced notions, and lay aside any ideas you may bring with you to this place, and take it for granted that we, your teachers, are trying honestly to bring you the truth, the latest, best, highest and most useful truth. It will be time enough when you set up for yourselves to compare what you have been taught here with what may be thought truth elsewhere.

I spoke, just now, of education as a drawing-out process. I trust that you will keep that in mind. We, who teach, are obliged to bear it in mind constantly. There is no teacher in all this university corps, or of any other university corps, who can make a great man out of you, or you, or you. A house may be built of bricks, but for the bricks you must, at least, have clay and fire too, and both are, by us, uncreatable. The brick is but another form of matter, so to speak, and we have drawn it out, educated it, out of the earth. So with our minds. We can only take the materials there and form, and fashion, and mold them into a certain shape they had not before. Certain elements, it is true, enter in, as the training of the eye, the training of the muscles and training of the thinking powers, which last we are fond, now-a-days, as fancying a sort of mental muscles. But the eye, the muscles, the brain must be there or there is no e-duco. Education is not, as seems commonly supposed, a sort of sausage-stuffing process, by virtue of which the empty student is stuffed to repletion with knowledge.

So, I conclude this part of the subject with the application of the old but rather coarse adage that one can not make a silk purse out of a swine's ear. And I trust there is no student who hears me who will expect more of this faculty than a strong helping hand and a deal of good advice, which last you may or may not follow.

In some colleges that I have seen, there seemed a strange spirit of wrath, almost, toward the Faculty. I am greatly in hope that there is none such here, and, indeed, I am glad to say that I have seen no such spirit here in the past. But I *have* seen students who seemed to think that what was the property of the college was a sort of lawful prey which they were privileged to break and destroy in a very uncivilized and savage fashion, as though they were a set of Goths or Vandals with a mission to smash the laboratory fixtures and destroy or injure the instruments. I trust that there is no such Goth or Vandal here, for, really, the things that are here are *yours*, not ours, in the sense that they are only useful to *you*. I dare say there is not a professor who would care to come here and while away his time in these laboratories if they were empty of students. They have something better to do. What is here is for *your* use, not ours, although the college furnishes it. These are the tools with which to do the "drawing out," and I hope I do not speak amiss when I appeal to the pride of every student here to see to it that the college-apparatus is as carefully handled by you as though you were trustees, accountable to a court.

I see before me a strangely mixed audience, as strange almost as confronted the Apostle Peter (I take it for granted you are, all of you, familiar with the history of the day of Pentecost) when he made his notable sermon on that occasion. I see the Caucasian of the West, the black man of the Eastern tropics, the yellow man of the Asiatic race assembled to-night representing three distinct races of mankind. But I see more still, for I see man and woman side by side, seeking knowledge from a source until very lately reserved to the man alone. It seems, even yet, with all our years of familiarity with this mingling of sexes a strange thing to see women studying professions for which, a few years ago, we thought them utterly unfit. But we become used to this, and it is proper we should when we find these same women, or their representatives, in the stores; in all places where light work is done, and where quick wits command a premium. To see these women in a class-room! It looks very shocking to some, and yet you, my friend, don't object to having a woman-nurse give you your medicine when you are sick; and please tell me why this dear woman shouldn't know how to compound the medicine, and know what sort of disease it is good for or even be able to discriminate the diseases when she sees them? The best doctor is a good nurse, and if you can combine both in one, and give the nurse the doctor's knowledge, is she any worse for it?

I remember, long ago, that men and women used to sit on opposite sides of the church; but that *was* long ago, when men didn't realize that women close at hand, like a magnet, were more powerfully attractive than at long range, and much more potent for good. And I would like, just here, to tell a bit of professional experience, in a teaching way: Some twenty years ago I was teaching in a college not a hundred miles from where we sit, a school with venerable associations, the oldest and, for years, the only dental college in the world. For years there had been young men only, and—disorder. How hard it is to keep the average young man student in order, only the village school-teacher and the college-professor can testify. But one session, at its beginning, the Faculty was confronted with a new and grave problem, albeit a pretty one. A young lady wished to study dentistry! She came from Germany; spoke good English; was well educated. But a woman in the class! The students heard of it and rose up in arms. "It should not be!" they said. They would make it hot for her! She would be glad to leave! Just let her try it!

The Faculty yielded to the fair speech of the fair German. She took a seat in the class-room, and a hush fell upon that class, such as was never known before. How that little German girl could so quickly leaven that great mass of disorder I never could comprehend, but she did it; and from that day the good behavior of the class was the talk of the Faculty-room. And I have to say that she passed a better examination than any of the students at "the final," too.

From that day no professor of that college has, so far as I am aware, objected to lady students. So much for sex in education; and now a single word as to color. We, of the Anglo-Saxon race, pride ourselves on our race. We have a feeling, right or wrong, that "we are the people," and that wisdom will die with us. This is classed as upstartishness by some, but I only want to say that unless a race has *race-pride* it can never rise to greatness. It is just as impossible for a race to grow great as it is for an individual, if the race or individual is simply aping some other successful race or nation. In INDIVIDUALITY alone lies the secret of success, and this is just as true of one race as of another. It takes, of course, only the most superficial observation to discover that no two persons have precisely the same views of things, and so no two nationalities are exactly alike; each must develop on its own lines. So I have to say to the different races represented here, that in trying to learn do not lay great stress on the fact that you can not do just what the workman of some other race does, just exactly in the same way he does it. Don't be afraid to be original; original in conception, in execution. In a word, be yourself. Being an Anglo-Saxon, I naturally feel that I am a little better than the rest of mankind who are not Anglo-Saxons. It is your privilege, nay, I am rather sure that it is your duty, to think *yourself* a little better than the other fellow. I want to say this, however, that no matter how I may esteem myself, and possibly overrate my own powers, for me to deny to the races in life of another color the privileges I claim for myself, would stamp me as unworthy of a place in the great brotherhood of man. I say I should be ashamed of myself and of my manhood were I to say, by any act of mine, "I can beat you in the race of life; I am sure of that, but I am going to arrange it so that you will not have an equal chance with me to run the race. I don't feel at all afraid that I will not succeed, but I am going to put stumbling-blocks in your way, you of the other race, lest *you* possibly might excel *me*." I say, to do this would be a confession of weakness truly pitiable. I am not only ready to encourage, but, if it were possible, I would give him whom I may, in my vanity, consider less capable than myself, even superior advantages.

In olden times, when the king was coming, a herald went before crying to the people, "The king cometh, clear the way for him." So I cry, not that the king comes, for here all are equal, all free; but the man who is trying to climb a little higher is coming; trying to climb up the ladder we have climbed. Let no one disturb the foundation of the ladder; let it stand on firm ground. To Faculty: Let us see to it that every round is strong, and the sides stout and solid. Help the man who has the ambition to climb. Give him your hand! On this ladder of education, fame—we who have achieved—have no right to place obstacles. If, in the providence of God, we are in any way ahead of him who now essays to rise, how mean, how utterly mean will we be if we do not not only clear the track, but cheer the racer. So at least it seems to me, and so I think, will the hearts of the people be.

And, after all, what is success? Is it in the making of money? It will be bad, indeed, if these students before me, when they start out in life, can not do this. But I beg you not to think that this is the end of life—its be-all and end-all. It is a good thing to have, and no man should be above its honest acquirement. But I do think, that for the professional man, there are aims so much higher than mere money-getting as not to be compared with it. If the man who made two blades of grass grow where but one grew before was considered a public benefactor, how much more should he considered a benefactor who takes away from a sick man his sicknesss? I conjure you, as men who are to study the fine art of healing, separate the operation, separate the *cure* from the *fee*, and having undertaken to do anything for any man, do this your very best, no matter whether the fee be small or large.

May I go on just far enough to say a word to those who are studying my specialty—dentistry? It is but a word. You have such peculiar advantages and facilities for the pursuit of your studies as should make you the envy of the medical student. For the medical student, no matter how thorough his course may be in this University, goes forth from its halls armed, it is true, with a diploma, but next to no practical knowledge of the profession. He must get his practice afterwards, and it is said, gets it at the expense of his patients, who have to suffer for his lack of practical skill. But with you, dental students, it is far different. You may be said to enter on the practice of your profession from the time you enter these walls. You are able to put in actual practice the teaching you receive, on the spot. You do not have to wait for your patients to come to you after you graduate, in order that you may know what to do; rather, day by day you have them here (possibly more's the pity for them), and many a man goes forth from the dental college thoroughly fitted in both hands and head for active, good work, work that will bring a competency.

